# Global, regional, and national impact of Down syndrome on child and adolescent mortality from 1980 to 2021, with projections to 2050: a cross-sectional study

**DOI:** 10.3389/fpubh.2025.1554589

**Published:** 2025-04-24

**Authors:** Erdengqieqieke Ye, Erman Wu, Rui Han

**Affiliations:** ^1^Department of Prenatal Diagnosis, Reproductive Medicine Center, The First Affiliated Hospital of Xinjiang Medical University, Urumqi, Xinjiang, China; ^2^Department of Neurosurgery, The First Affiliated Hospital of Xinjiang Medical University, Urumqi, Xinjiang, China; ^3^Department of Computer Science and Information Technologies, Elviña Campus, University of A Coruña, A Coruña, Spain; ^4^Xinjiang Clinical Research Centre for Reproductive Immunology, The First Affiliated Hospital of Xinjiang Medical University, Urumqi, Xinjiang, China

**Keywords:** Down syndrome, global disease burden, mortality, estimated annual percentage change, projections

## Abstract

**Introduction:**

Down syndrome, resulting from trisomy 21, is a prevalent genetic disorder. Despite improvements in life expectancy and quality of life due to medical progress, children and adolescents (under the age of 20 years) with Down syndrome still face higher mortality rates. Future research is essential to elucidate the epidemiological patterns and trends in Down syndrome among children and adolescents, enabling the development of effective prevention and intervention strategies to improve survival and health outcomes.

**Methods:**

This study draws on Global Burden of Disease (GBD) 2021 mortality data for children and adolescents with Down syndrome. Pearson’s correlation coefficient was leveraged to assess the relationship between Down syndrome mortality and the Socio-demographic Index (SDI). The estimated annual percentage change (EAPC) in mortality was calculated to track temporal trends, and the Bayesian age-period-cohort (BAPC) model was employed to forecast future mortality.

**Results:**

Over the past 42 years, there have been fluctuations in mortality among children and adolescents with Down syndrome. Globally, deaths have decreased by 22.8% from 26.95 thousand (95% uncertainty interval [UI], 10.10–74.66 thousand) in 1980 to 20.81 thousand (95% UI, 14.18–36.49 thousand) in 2021. Furthermore, BAPC model projections indicate a sustained reduction in mortality for children and adolescents with Down syndrome. Predominantly, deaths occur in 0–4 age group, with higher death rates in Low SDI regions, and notably, the number and rate of female patients exceed those of male patients. Intriguingly, a negative correlation was observed between death rates and higher SDI.

**Conclusion:**

Most countries have seen a decline in Down syndrome deaths among children and adolescents over the last 42 years, but a few high SDI countries are witnessing an increase. Future health interventions should prioritize these countries, focusing on resource allocation, infrastructure, and health education. Continued efforts on care for the 0–4 age group with Down syndrome are crucial to further reducing deaths in this age group.

## 1 Introduction

Down syndrome, also known as trisomy 21 syndrome, is a common congenital birth defect that results from chromosomal abnormalities (1, 2). Typically, this condition occurs when an individual has an extra copy of chromosome 21, leading to a total of 47 chromosomes instead of the usual 46 (1, 3). This additional chromosome 21 triggers a variety of characteristic clinical features, such as intellectual disability, congenital heart disease, immune system dysfunction, and digestive system abnormalities (4–8). Roughly half of the babies born with Down syndrome have congenital heart defects. The most common types are holes in the walls that separate the heart’s chambers, known as atrioventricular septal defect (42%), ventricular septal defect (22%), and atrial septal defect (16%) (9). Over the course of time, a significant reduction has been observed in the incidence of severe congenital heart defects among infants diagnosed with Down syndrome (9). Children with Down syndrome exhibit a 10–20-fold higher incidence of acute leukemia, with acute megakaryoblastic leukemia (AMKL) being notably more prevalent (10). This form of leukemia is associated with an estimated 500-fold increased relative risk when compared to the general population (10). Furthermore, individuals with Down syndrome are more prone to pneumonia and serve respiratory infection than the general population (11). This susceptibility significantly affects their health, often necessitating specialized care and extended hospital stays (12). These complications remain a significant contributor to mortality among the population with Down syndrome (11, 13–15). Consequently, early detection and intervention are crucial for reducing morbidity and mortality among individuals with Down syndrome (16, 17).

Individuals with Down syndrome generally experience a reduced life expectancy in comparison to the general population (18). Notably, mortality rates are elevated among young adults with Down syndrome, particularly in their 20s, with heightened vulnerability observed in females (19). However, there is a lack of comprehensive research examining the mortality patterns of children and adolescents across various regions and age groups. Comprehending the mortality patterns and future projections for children and adolescents with Down syndrome at global, regional and national levels is essential. It enables more effective resource distribution, informs the creation of focused public health strategies, and allows for the assessment of interventions (20, 21). This knowledge also raises public awareness, guide research, and fosters international cooperation. Ultimately, it aids in planning for future healthcare needs, ensuring preparedness and quality care for individuals with Down syndrome.

Recent research has concentrated on the disease burden of Down syndrome from 1990 to 2021, analyzing Years Lived with Disability (YLDs), Years of Life Lost (YLLs), Disability-Adjusted Life Years (DALYs) using data from the Global Burden of Disease 2019 study (22, 23). Additionally, recent study has utilized GBD 2021 data to assess the disease burden of Down syndrome from 1990 to 201, with projection to 2040 (24). Currently, no studies have comprehensively examined mortality among children and adolescents with Down syndrome, and there has been scant attention to forecasting future trends. Yet, this study systematically analyzes mortality data from 1980 to 2021, including regional, gender and SDI disparities among children and adolescents, and projects the trends to 2050.

## 2 Methods

### 2.1 Data source

The GBD 2021 study by the Institute for Health Metrics and Evaluation (IHME) at the University of Washington offers a thorough analysis of global health burden from disease and injuries^1^ (25). Down syndrome mortality data from the GBD 2021, spanning from 1980 to 2021, were extracted for worldwide, five SDI regions, 21 GBD areas and 204 countries and territories. In accordance with the IHME definition of children and adolescents (26), data for Down syndrome individuals aged less than 5 years, 5–9 years, 10–14 years, and 15–19 years were aggregated into a single category of 0–19 years, representing the dataset for children and adolescents with Down syndrome.

In the 10th edition of the International Classification of Diseases (ICD-10), Down syndrome is categorized under the codes Q90-Q90.9. Under the 9th revision of the International Classification of Diseases (ICD-9), Down syndrome is assigned to the code 758.0.

### 2.2 Statistical analysis

The Estimated Annual Percentage Change (EAPC), a key metric for tracking the Age-standardized Rate (ASR) progression. The model is expressed as y = *α* + βx + *ε*, where y is the annual rate of change per 100,000 people, αis the intercept, *β*is the slope coefficient, x denotes the calendar year, and εis the error term. The EAPC is derived from the formula:


$$\mathrm{EAPC}=100^{*}\;\left(\exp\left(\beta \right)–1\right)$$

Confidence intervals at the 95% level are directly from the regression model. An alpha level of 0.05 is used to determine statistical significance for two-sided tests.

Concurrently, the Pearson correlation coefficient assesses the correlation between ASR and SDI, with significance set at *p*-values less than 0.001.

These analyzes were performed utilizing R software, version 4.4.1.

### 2.3 Projection analysis

We adopted the Bayesian Age-Period-Cohort (BAPC) R package for Bayesian Age-Period-Cohort modeling to predict future disease burden (27). Based on age-specific population data from 1980 to 2021 and projected population data from 2022 to 2050, we evaluated long-term mortality trends. The BAPC model addresses parameter non-identifiability by imposing constraints and employs 5th-degree B-splines to smoothly model age and period effects. Additionally, intrinsic Gaussian Markov Random Field priors were applied to enforce local correlations between adjacent effects. Posterior inference was performed via Markov Chain Monte Carlo sampling, and the stability of predictions was assessed using leave-one-out cross-validation.

## 3 Results

### 3.1 Trends in mortality of Down syndrome in children and adolescents from 1980 to 2021 and projections to 2050

In 2021, the estimated number of global deaths among children and adolescents with Down syndrome stood at 20.81 thousand (95% UI, 14.18–36.49), which signifies a substantial 22.8% decrease from the 26.95 thousand deaths (95% UI, 10.10–74.66) that were documented in 1980. This reduction highlights a positive trend in mortality rates over the past four decades. Concurrently, the global death rate for children and adolescents with Down syndrome in 2021 was 0.79 per 100,000 people, a significant decrease from the 1.31 per 100,000 people observed in 1980 (95% UI, 0.49–3.64) (Table 1).

**Table 1:** Mortality trends of Down syndrome in children and adolescents from 1980 to 2021 by geographic region.

Characteristics	1980		2021		1980–2021
	The number of deaths (95% UI)	Death rates (95% UI)	The number of deaths (95% UI)	Death rates (95% UI)	EAPC of death rates (95% CI)
Global	26.95 (10.1–74.66)	1.31 (0.49–3.64)	20.81 (14.18–36.49)	0.79 (0.54–1.38)	−1.08 (−1.18–0.98)
Female	14.52 (3.78–43.87)	1.45 (0.38–4.38)	10.77 (6.93–22.03)	0.84 (0.54–1.72)	−1.08 (−1.18–0.98)
Male	12.43 (5.3–37.09)	1.19 (0.51–3.54)	10.04 (6.77–16.97)	0.74 (0.5–1.25)	−1.07 (−1.17–0.97)
Low SDI	6.56 (1.43–22.23)	2.99 (0.65–10.12)	9.58 (5.15–21.62)	1.64 (0.88–3.7)	−1.09 (−1.19–0.99)
Low-middle SDI	6.87 (1.89–22.42)	1.37 (0.38–4.47)	5.51 (3.78–8.76)	0.72 (0.49–1.15)	−1.22 (−1.32–1.12)
Middle SDI	7.86 (3.1–19.43)	1.13 (0.44–2.79)	3.79 (3.07–4.78)	0.51 (0.41–0.64)	−1.94 (−2.1–1.79)
High-middle SDI	4.56 (2.27–9.17)	1.23 (0.62–2.48)	1.27 (0.96–1.57)	0.42 (0.32–0.52)	−2.66 (−2.87–2.46)
High SDI	1.08 (0.84–1.63)	0.41 (0.32–0.62)	0.63 (0.51–0.73)	0.27 (0.22–0.32)	−1.01 (−1.16–0.86)
Australasia	0.01 (0.01–0.02)	0.2 (0.17–0.24)	0.02 (0.02–0.03)	0.32 (0.23–0.42)	1.76 (1.37–2.16)
Oceania	0.07 (0.01–0.19)	2.55 (0.47–6.98)	0.25 (0.07–0.49)	3.95 (1.11–7.63)	1.31 (1.18–1.43)
East Asia	5.26 (2.2–11.15)	1.12 (0.47–2.37)	1.38 (0.73–1.96)	0.4 (0.21–0.57)	−2.92 (−3.32–2.51)
Central Asia	0.22 (0.1–0.45)	0.79 (0.37–1.61)	0.15 (0.1–0.22)	0.43 (0.3–0.65)	−1.16 (−1.28–1.04)
South Asia	4.3 (1.06–16.64)	0.94 (0.23–3.62)	2.52 (1.43–4.88)	0.37 (0.21–0.71)	−2.02 (−2.13–1.92)
Southeast Asia	1 (0.3–3)	0.5 (0.15–1.5)	0.97 (0.71–1.35)	0.42 (0.31–0.59)	−0.2 (−0.29–0.12)
High-income Asia Pacific	0.28 (0.21–0.45)	0.51 (0.39–0.82)	0.05 (0.04–0.07)	0.17 (0.13–0.21)	−2.6 (−3.17–2.03)
Eastern Europe	0.53 (0.43–0.68)	0.81 (0.67–1.04)	0.09 (0.07–0.12)	0.2 (0.15–0.27)	−3.6 (−3.82–3.37)
Central Europe	0.14 (0.09–0.24)	0.36 (0.24–0.62)	0.04 (0.03–0.05)	0.18 (0.13–0.22)	−1.24 (−1.46–1.01)
Western Europe	0.53 (0.46–0.64)	0.48 (0.41–0.57)	0.3 (0.24–0.35)	0.33 (0.26–0.38)	−0.31 (−0.64–0.02)
High-income North America	0.18 (0.16–0.21)	0.22 (0.2–0.26)	0.17 (0.13–0.2)	0.19 (0.15–0.22)	0.1 (−0.08–0.29)
Andean Latin America	0.61 (0.18–1.42)	3.78 (1.09–8.75)	0.23 (0.17–0.32)	0.98 (0.72–1.36)	−3.2 (−3.33–3.06)
Central Latin America	0.64 (0.44–0.84)	0.88 (0.61–1.16)	0.61 (0.44–0.84)	0.72 (0.52–0.99)	−0.62 (−0.82–0.41)
Southern Latin America	0.25 (0.18–0.35)	1.45 (1.03–2.02)	0.13 (0.1–0.17)	0.69 (0.53–0.9)	−0.71 (−1–0.42)
Tropical Latin America	0.84 (0.45–1.22)	1.33 (0.72–1.94)	0.43 (0.33–0.57)	0.65 (0.5–0.86)	−0.77 (−1.04–0.5)
Caribbean	0.21 (0.08–0.67)	1.44 (0.57–4.49)	0.18 (0.08–0.44)	1.21 (0.53–2.86)	−0.64 (−0.82–0.46)
North Africa and Middle East	5.78 (0.99–19.92)	4.13 (0.71–14.23)	2.81 (1.94–4.53)	1.19 (0.82–1.91)	−2.66 (−2.78–2.54)
Eastern Sub-Saharan Africa	2.53 (0.37–9.49)	3.08 (0.45–11.55)	3.49 (1.81–7.56)	1.53 (0.8–3.32)	−1.67 (−1.77–1.56)
Central Sub-Saharan Africa	0.64 (0.12–2.46)	2.68 (0.5–10.27)	1.09 (0.51–2.47)	1.49 (0.69–3.36)	−1.05 (−1.28–0.83)
Southern Sub-Saharan Africa	0.29 (0.15–0.53)	1.34 (0.71–2.47)	0.38 (0.24–0.52)	1.2 (0.76–1.66)	−0.09 (−0.35–0.18)
Western Sub-Saharan Africa	2.64 (0.63–7.4)	3.25 (0.78–9.11)	5.5 (2.82–12.83)	2.05 (1.05–4.78)	−0.15 (−0.35–0.04)

**Figure 1:**
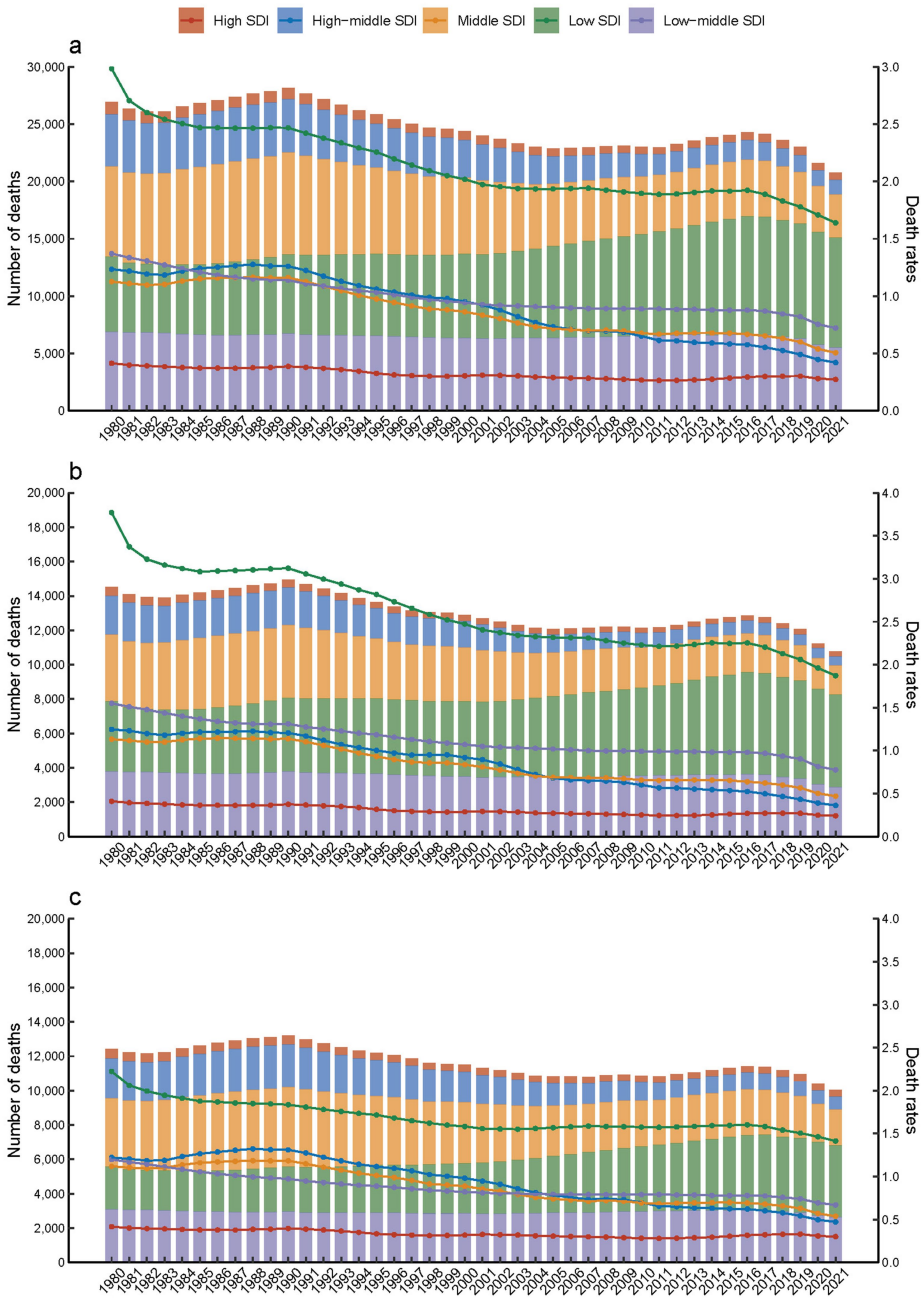
Down syndrome mortality in children and adolescents by SDI quintiles from 1980 to 2021. **(a)** Both, **(b)** females, **(c)** males.

Furthermore, in 2021, it was observed that children and adolescents with Down syndrome in regions with low SDI had the highest death rates compared to those in other SDI categories (Figure 1a). In contrast, deaths in low SDI areas increased from 6.56 thousand (95% UI, 1.43–22.23) in 1980 to 9.58 thousand (95% UI, 5.15–21.62) in 2021 (Table 1). Additionally, in 2021, there was a slightly higher mortality rate among females with Down syndrome, at 0.84 per 100,000 (95% UI, 0.54 to 1.72), compared to males, which was 0.74 per 100,000 (95% UI, 0.5 to 1.25) (Figures 1b,c; Table 1).

**Figure 2:**
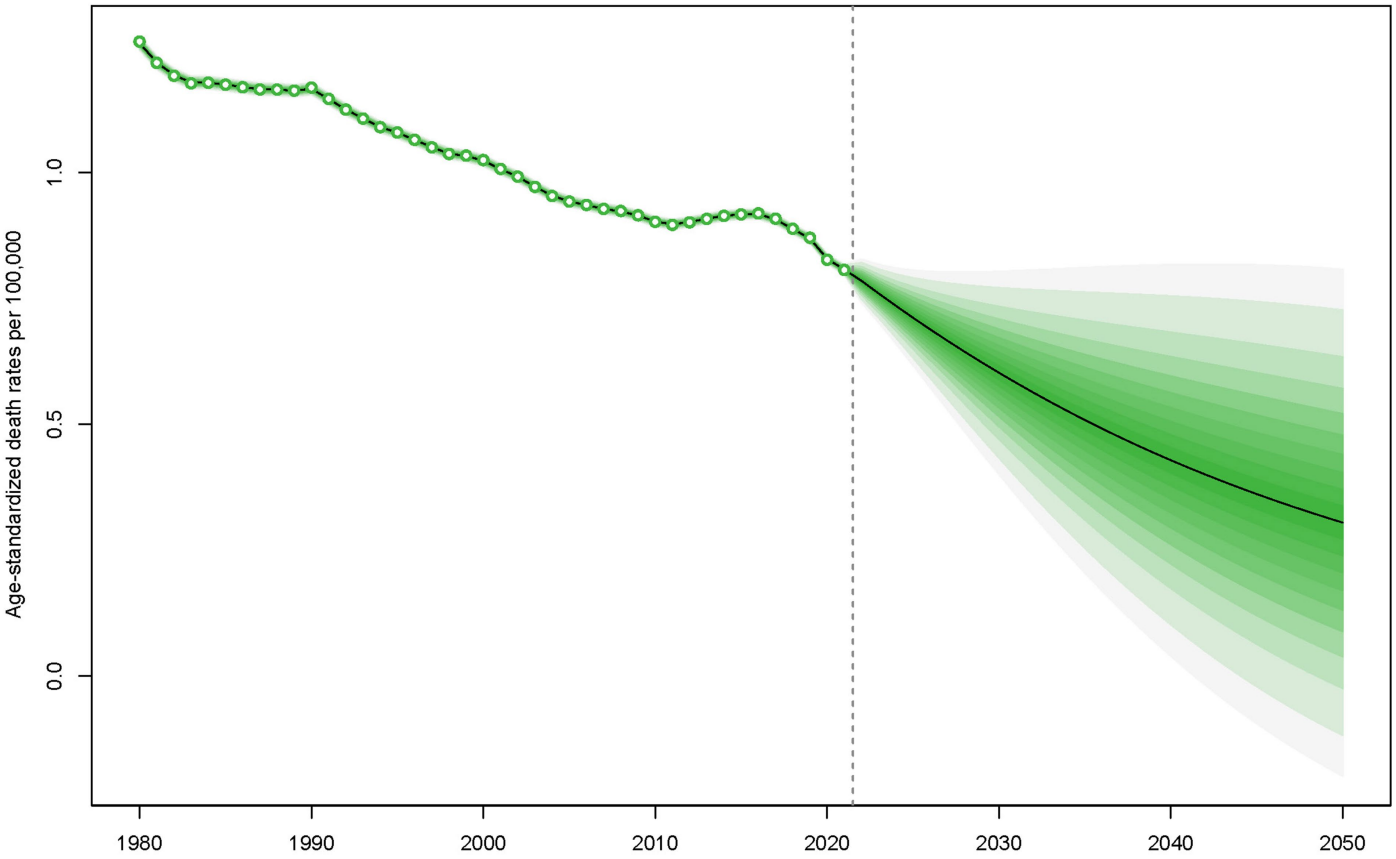
Forecasted mortality rates for children and adolescents with Down syndrome from 2022 to 2050.

Employing the Bayesian Age-Period-Cohort (BAPC) model, projections of mortality rate trends for children and adolescents with Down syndrome from 2022 to 2050 have been conducted. The predictions suggest that a continued decline in mortality rates is anticipated between 2022 and 2050, with a significant reduction foreseen (Figure 2).

### 3.2 Age-specific mortality rates of Down syndrome in children and adolescents from 1980 to 2021

In accordance with the five SDI and 21 GBD regions, children and adolescents with Down syndrome were categorized into four distinct age brackets: 0–4 years, 5–9 years, 10–14 years, and 15–19 years.

**Figure 3:**
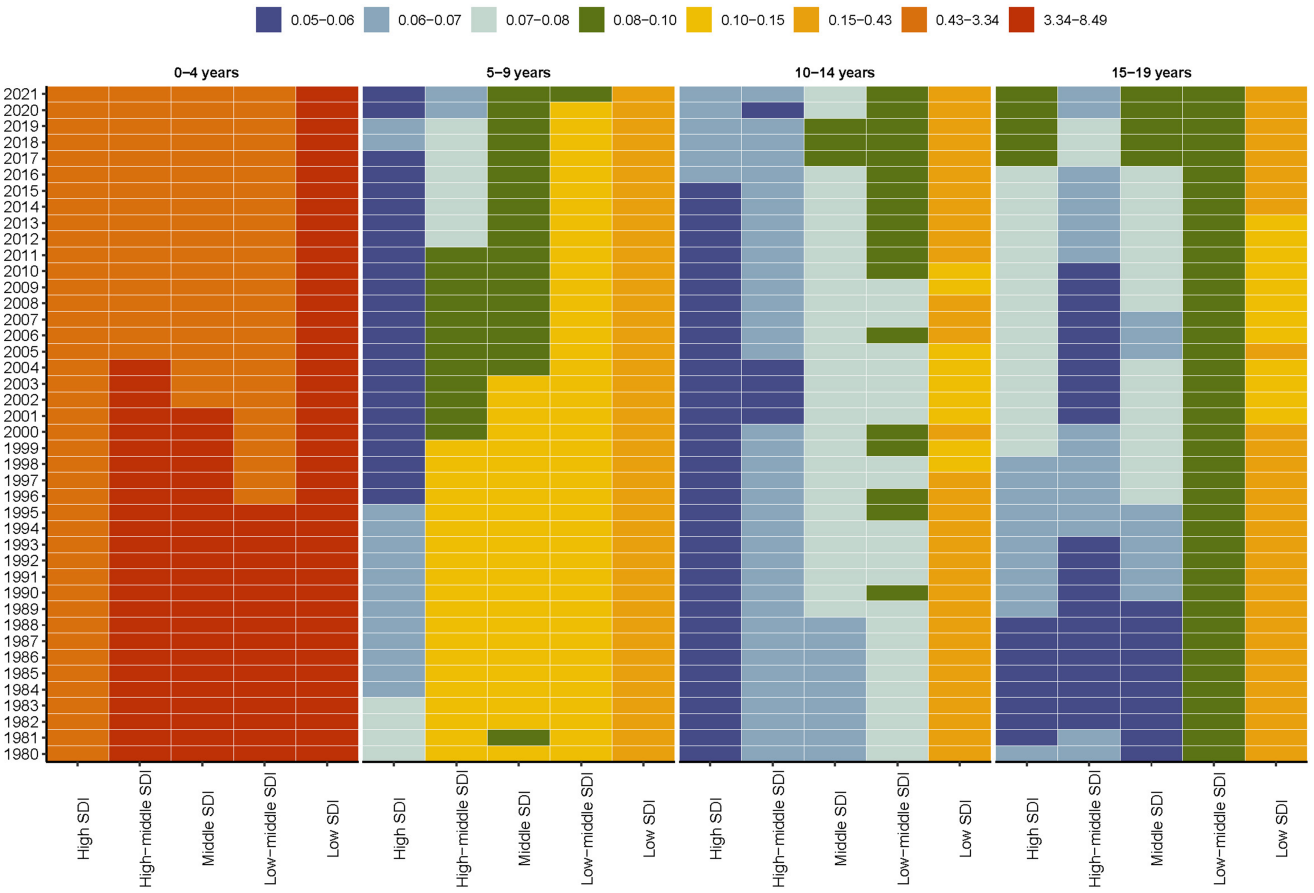
Global age-stratified mortality rates for Down syndrome in children and adolescents by SDI quintiles, 1980–2021.

This stratification allowed for a comprehensive analysis of mortality rate across different age groups over a 42-year period, spanning from 1980 to 2021. Specifically, children with Down syndrome in the 0–4 years age groups exhibited a significantly higher mortality rate compared to other age groups. Furthermore, it is noteworthy that across all age groups, the mortality rate for individuals with Down syndrome remained consistently elevated in regions classified as low SDI throughout the entire duration of the study, which spanned from 1980 to 2021 (Figure 3). Notably, a recent trend has emerged in high SDI regions, where the mortality rate for individuals with Down syndrome in the 10–14 years and 15–19 years age groups has shown an upward trend (Figure 3).

**Figure 4:**
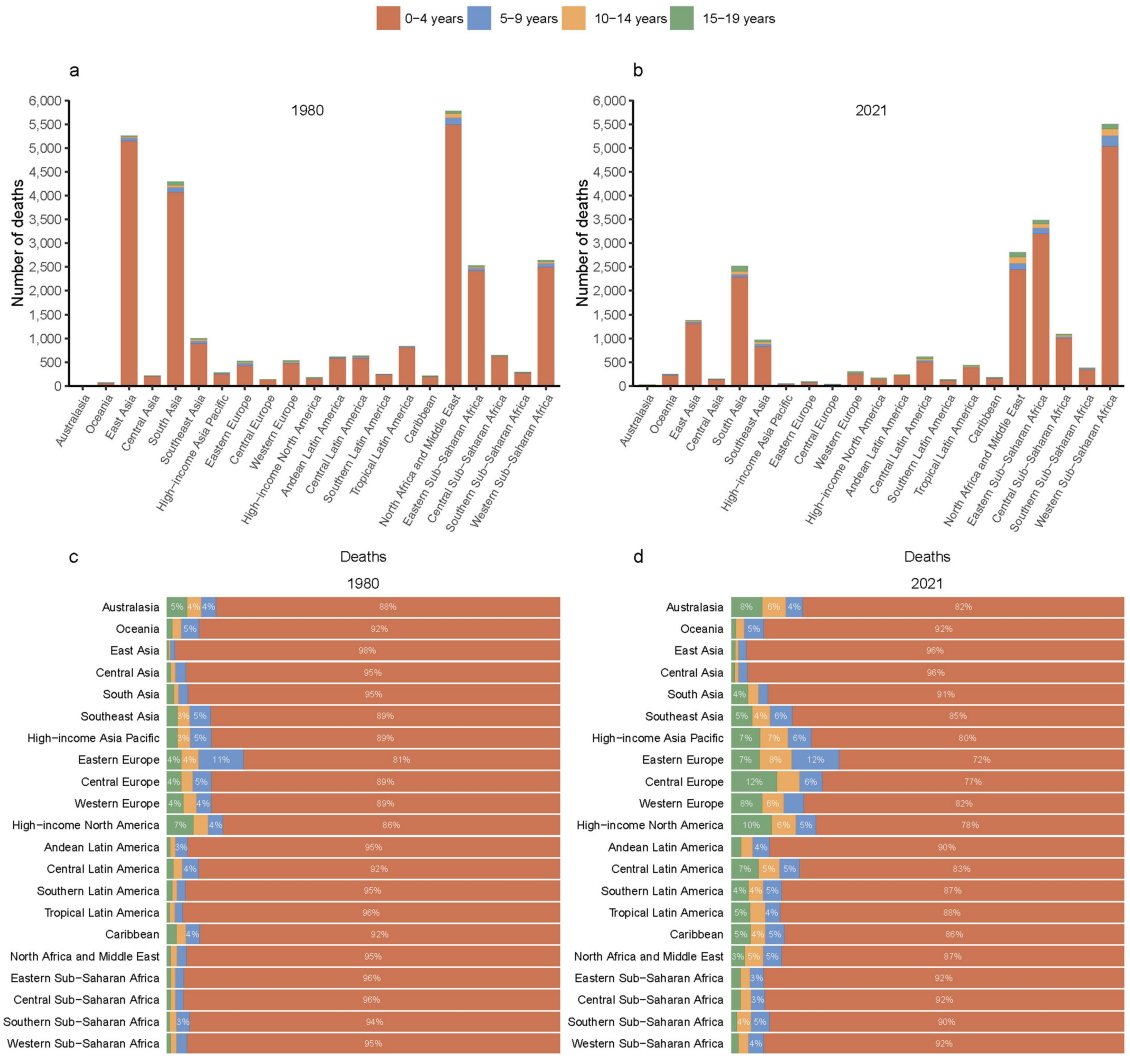
Mortality from Down syndrome among children and adolescents across 21 GBD regions in 1980 **(a)** and 2021 **(b)**, along with proportional distributions in 1980 **(c)** and 2021 **(d)**.

While East Asia, South Asia, and North Africa and Middle East have experienced substantial reductions in the deaths of children and adolescents with Down syndrome since 1980, it is particularly striking to observe the decline in deaths in these regions (Figures 4a,b). Specifically, the mortality figures for East Asia, South Asia, and the North Africa and Middle East have dropped from 5.26 thousand (95% UI, 2.2–11.15 thousand), 4.3 thousand (95% UI, 1.06–16.64 thousand), 5.78 thousand (95% UI, 0.99–19.92 thousand) to 1.38 thousand (95% UI, 0.73–1.96 thousand), 2.52 thousand (95% UI, 1.43–4.88 thousand) and 2.81 thousand (95% UI, 1.94–4.53 thousand) respectively (Table 1). In contrast, Western Sub-Saharan Africa has witnessed an increase in deaths among this demographic, rising from 2.64 thousand (95% UI, 0.63–7.4 thousand) to 5.5 thousand (95% UI, 2.82–12.83 thousand) (Figures 4a,b; Table 1).

Significantly, the analysis revealed that the 0–4 years age group continues to account for the majority of Down syndrome-related deaths in 21 GBD regions. From 1980 to 2021, there was a decrease in the proportion of the 0–4 years age group in nearly all GBD regions (Figures 4c,d).

### 3.3 Association of mortality rate and SDI in Down syndrome children and adolescents in 2021

**Figure 5:**
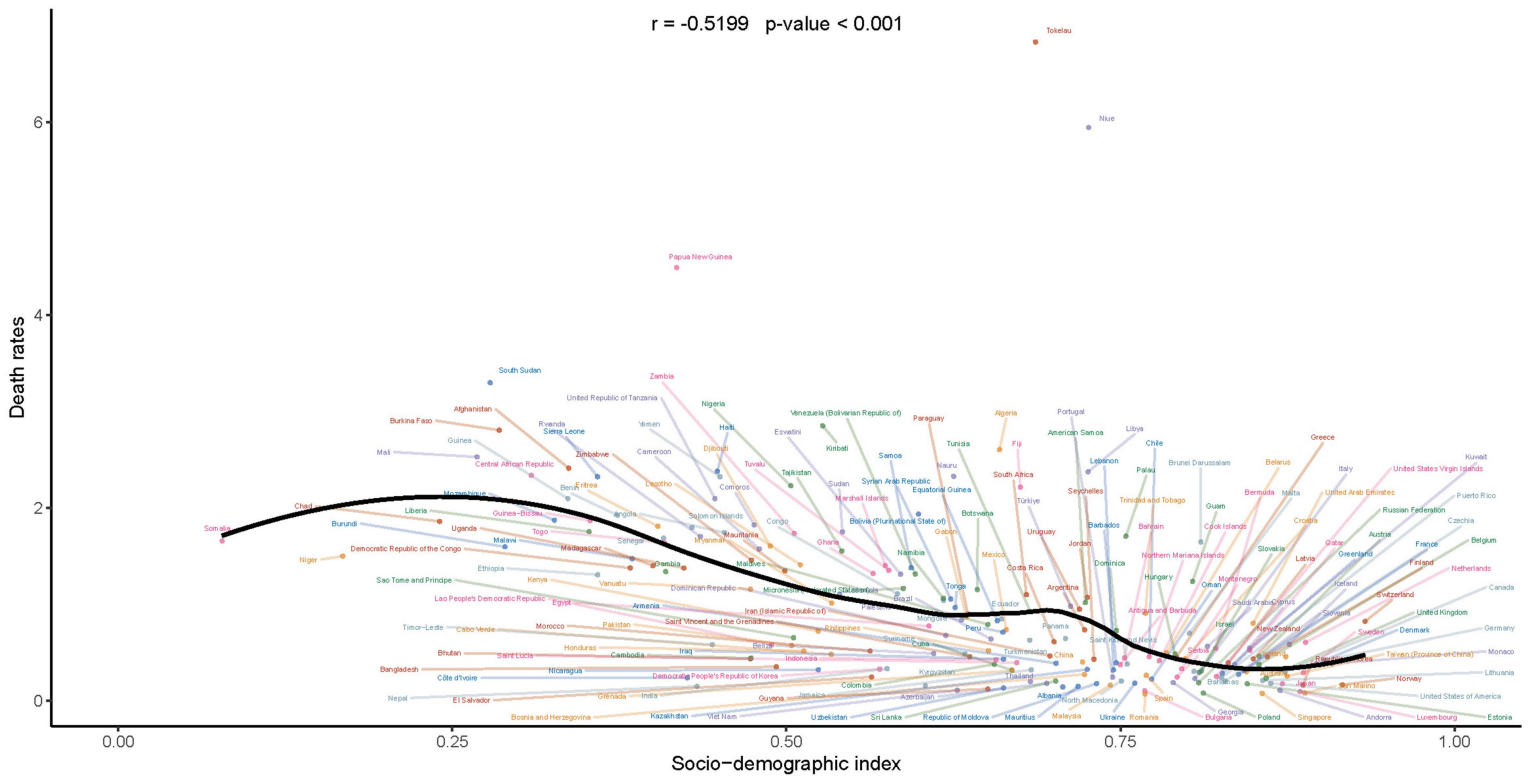
Correlation of mortality rates with socio-demographic index in children and adolescents with Down syndrome.

Upon conducting a meticulous analysis of the mortality rates associated with Down syndrome in children and adolescents, we observed a discernible trend: as the SDI increases, there is a significant negative correlation with mortality rates. This correlation reveals that a higher socio-economic status is inversely linked to health outcomes for children and adolescents with Down syndrome. Consequently, it suggests that enhanced social and economic conditions are associated with improved survival rates within this specific demographic (Figure 5). The robustness and relevance of this inverse relationship are further emphasized by the correlation coefficient of −0.5199, indicating a moderate to strong negative correlation. This means that an elevated SDI correlates with reduced mortality rates among children and adolescents with Down syndrome. Moreover, the statistical significance of this correlation is affirmed by the *p*-value, which is below 0.001.

### 3.4 Mortality trends in children and adolescents with Down syndrome by countries

**Figure 6:**
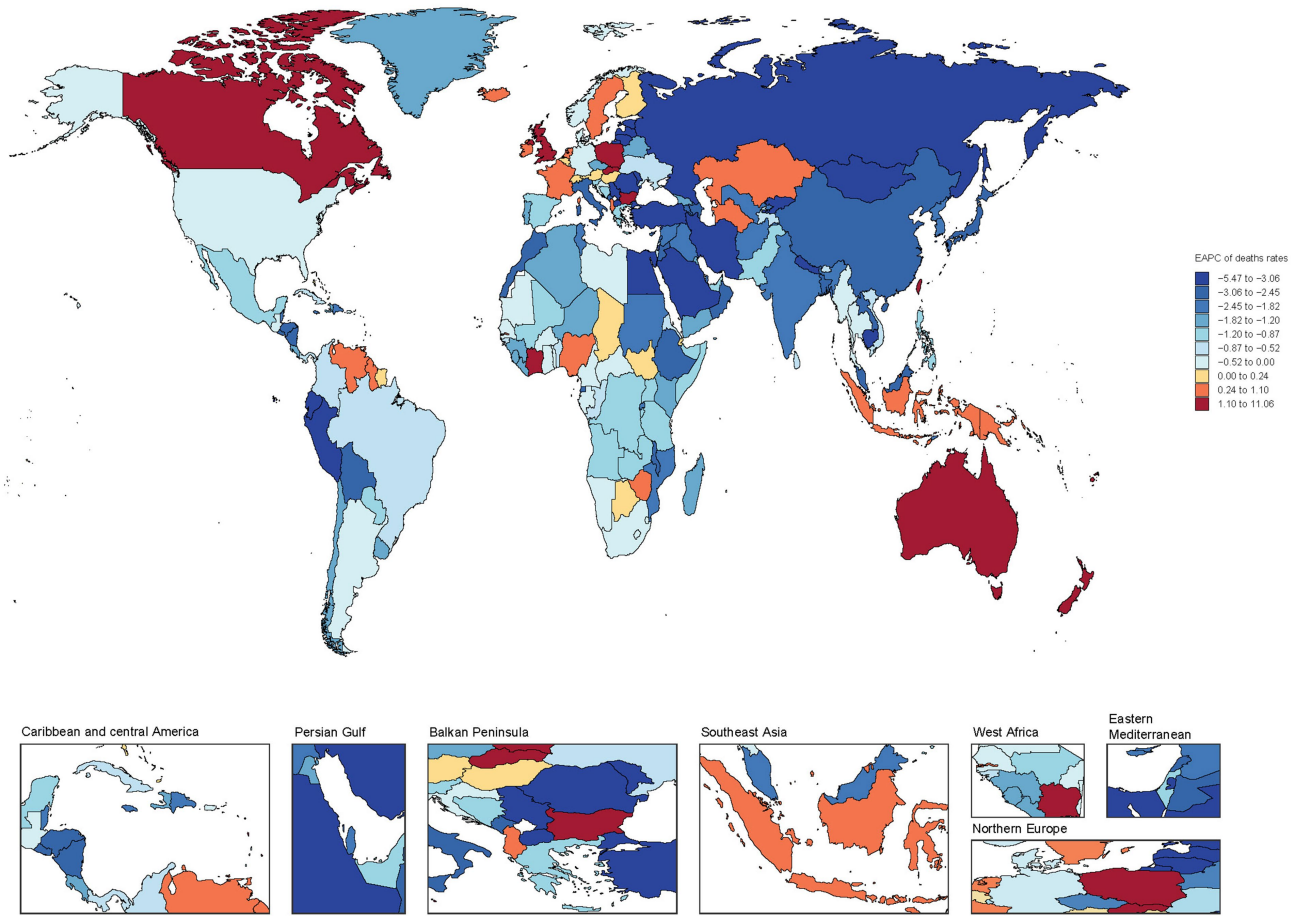
Global map of EAPC in mortality rates for children and adolescents with Down syndrome.

Globally, a predominant pattern of decline is observed in the EAPC of mortality rates for children and adolescents with Down syndrome across the majority of countries and regions. However, it is noteworthy that a minority of countries and regions have exhibited an upward trend in the EAPC of mortality rates for this population (Figure 6). Within this context, a significant observation is the increase in EAPC for Down syndrome mortality among children and adolescents in several high-SDI countries. For instance, Poland has witnessed an EAPC of 10.58 (95% CI, 8.95–12.22), the United Kingdom at 7.66 (95% CI, 6.9–8.44), New Zealand at 3.48 (95% CI, 2.69–4.27), Australia at 1.42 (1.06–1.78), and Canada at 1.27 (95% CI, 1.07–1.48) for Down syndrome mortality rates (Supplementary Table S1). These figures underscore the complexity of the global landscape regarding Down syndrome mortality, where despite a general downward trend, certain high-SDI nations have experienced an unexpected increase in EAPC of mortality rate.

## 4 Discussion

This research delves into the mortality rate of Down syndrome in the pediatric and adolescent population, extending its scope to forecast mortality rates up to the year 2050. Although previous studies have examined the disease burden that Down syndrome imposes on affected individuals (22–24), they have not specifically focused on its impact on children and adolescents, nor have they ventured to predict future trends extending to the year 2050. This study aims to fill that gap by providing a comprehensive analysis of the current state of Down syndrome mortality and its projected trend over the coming decades.

In this study, from 1980 to 2021, global deaths among children and adolescents with Down syndrome decreased by 22.8%, driven by a significant reduction in regions like East Asia, South Asia, and the North Africa and Middle East (Table 1; Figures 1, 4). This reduction can be partially credited to enhancements in medical care and heightened prevention strategies (28–30). In these regions, better healthcare conditions, advancements in diagnostics, and more effective management of complications associated with Down syndrome have contributed to increased life expectancy and a consequent drop in mortality rates (31, 32).

These reductions are projected to continue through 2050, as indicated by the BAPC model (Figure 2). However, despite this overall trend, regions with low SDI still experience higher mortality rates (Figure 1). Additionally, while the 0–4 age group saw the highest mortality rates (Figure 3). A pattern driven by a complex interplay of biological and systemic factors. Congenital anomalies—particularly cardiac defects combined with heightened susceptibility to infections due to immune dysregulation, create a critical vulnerability window during early childhood (33, 34). Compounding these risks, treatment-related complications and delayed access to specialized neonatal care in low-resource settings further exacerbate outcomes (35, 36). Concurrently, policymakers should address structural inequities by investing in healthcare infrastructure, training community health workers, and implementing telemedicine initiatives to bridge gaps in early diagnosis and treatment accessibility for this high-risk population. The ongoing decline in mortality rates for children and adolescents with Down syndrome is a reflection of the continuous improvements in disease management and implementation of prevention measures (31, 37). This analysis highlights the critical need for sustained monitoring and intervention strategies to further decrease mortality rates, especially in low SDI regions where the impact of Down syndrome remains substantial. In these areas, limited medical resources and undeveloped healthcare systems lead to difficulties in accessing medical services and treatment, and thus experiencing higher mortality rates (38). Furthermore, it indicates that within the 0–4 age group, there is still a higher mortality rate despite the general decline. This could be attributed to the physiological vulnerabilities and increased susceptibility of young children in this age group to infections and complications (39–41).

Our analysis reveals a notable gender disparity in mortality rates among individuals with Down syndrome, with female patients experiencing significantly higher mortality compared to males. This pattern may be partially explained by biological vulnerabilities, as females with Down syndrome exhibit a higher prevalence of severe congenital anomalies—particularly atrioventricular septal defects (AVSDs), and substantially elevate risks of early-onset heart failure (18, 42). Beyond biological factors, socio-cultural dynamics further exacerbate these disparities: systemic gender biases in healthcare access disproportionately disadvantage females with Down syndrome, who are less likely to receive timely cardiac interventions or preventive care compared to males, particularly in low-resource settings (43). These intersecting risks underscore the urgent need for gender-sensitive clinical protocols, including prioritized cardiac screening for female neonates and community-based programs to address caregiving biases. Addressing these gaps could mitigate preventable deaths and advance equity in Down syndrome care.

Even though higher SDI is typically linked to a decrease in mortality rates for children and adolescents with Down syndrome, it’s important to note that some regions with high SDI have seen a worrying increase in mortality rates among the 10–19 age groups (Figures 2, 4). With the general enhancement in survival rates for individuals with Down syndrome, they are likely to encounter health issues related to aging at a younger age, such as early-onset Alzheimer’s disease, cardiovascular disease, and immune system challenges. These conditions tend to be more severe among adolescents and young adults (38). In addition to these health concerns, adolescents and young adults might also be exposed to a range of social and behavioral risk factors, including unhealthy lifestyles, poor dietary habits, and a heightened risk of accidental injuries (39). These risks are not exclusive to low SDI areas but can also be present in high SDI regions, potentially affecting individuals with Down syndrome. Moreover, there may be variations in the availability and quality of data concerning adolescent mortality (1).

Moreover, while the expected annual percentage change (EAPC) in mortality rates for young people with Down syndrome generally shows a downward trend, a few high-SDI nations, including Poland, the United Kingdom, New Zealand, Australia, and Canada, have paradoxically experienced an unexpected rise in EAPC for Down syndrome-related deaths. This highlights the complexity of the relationship between socio-economic development and health outcomes in the context of Down syndrome. First, evolving medical practices—such as perioperative care protocols for congenital heart defects—may inadvertently elevate risks in adolescents due to delayed complications or inadequate long-term management (44). Second, inconsistencies in mortality data classification (e.g., misattributing Down syndrome as a contributing rather than primary cause of death) obscure true epidemiological patterns, potentially masking systemic gaps in care (45). Third, environmental factors like air pollution may synergize with the immunodeficiencies inherent to Down syndrome, exacerbating susceptibility to infections and chronic conditions in adolescents (46). Typically, countries with high SDI boast superior medical infrastructure and more favorable living conditions, which are expected to lead to reduced mortality rates (16).

However, the observed increase in deaths related to Down syndrome in these countries suggests that other factors may be at play. These could include genetic predispositions, environmental influences, shifts in medical practices, or alterations in data recording methodologies. This underscores an urgent need for additional research to decipher the causes behind these trends, thereby equipping policymakers and healthcare providers with the insights necessary to enhance the quality of life and health outcomes for individuals with Down syndrome.

This study offers an in-depth analysis of the mortality rate of Down syndrome in children and adolescents from 1980 to 2021, with forecasts extending to 2050. However, there are a few limitations to consider. First, the study does not include data beyond 2021, which is crucial given the potential effects of the COVID-19 pandemic on health and mortality for individuals with Down syndrome. This gap is particularly significant given recent findings that individuals with Down syndrome faced a mortality risk from COVID-19 ten times greater than the general population (47). Updated data could shed light on medical interventions, resource availability, and the pandemic’s impact on this population (48, 49). Second, the study did not pinpoint the precise causes of death associated with Down syndrome, such as fatalities due to congenital heart disease, leukemia, or other related health conditions. For instance, Dimopoulos et al. demonstrated that congenital heart disease contributes to approximately 50% of mortality in individuals with Down syndrome, while Hitzler et al. identified a 20-fold increased risk of acute lymphoblastic leukemia in this population compared to the general cohort. These findings highlight the critical need for future studies to prioritize the inclusion of cause-specific mortality data in individuals with Down syndrome, as such granular analysis is essential for elucidating disease mechanisms, informing clinical management strategies, and ultimately improving health outcomes in this vulnerable population (14, 15, 18). Lastly, to confirm these findings and better understand how socioeconomic development influences health outcomes for those with Down syndrome, long-term, multi-regional studies are essential.

Future research efforts should concentrate on understanding the impact of Down syndrome in low SDI regions or countries, where the mortality of the condition is typically higher. These measures may help improve the health status of individuals with Down syndrome in low SDI regions. (1) Strengthening primary care via mobile clinics and training local workers for early diagnosis. (2) Allocating resources to WHO—endorsed programs, subsidized drugs, and diagnostics. (3) Implementing simplified disease registries with telemedicine and low—cost tools. Additionally, studies should investigate context-specific strategies for improving healthcare delivery, including: (1) prioritizing resource allocation in low-resource settings (particularly middle-income countries) through mobile clinics and subsidized diagnostics; (2) enhancing mortality data standardization in regions with established healthcare infrastructure; and (3) developing age-specific interventions for the critical 0–4 age cohort, such as mandatory congenital heart defect screening and caregiver competency training programs. These evidence-based strategies should aim to translate epidemiological findings into targeted policies that reduce survival disparities across socioeconomic settings while maintaining focus on middle-income country implementation.

In summary, we document a significant global decrease in mortality rates for children and adolescents with Down syndrome over a 42-year period. Our findings reveal that the 0–4 age group experiences the highest mortality, predominantly in regions with low Socio-demographic Index (SDI). We also observe a higher mortality rate among female patients compared to males. Furthermore, our analysis indicates a negative correlation between mortality rates and higher SDI, suggesting that increased socio-economic development is associated with lower mortality rates for individuals with Down syndrome.

## Data Availability

All data used in this research are publicly at https://vizhub.healthdata.org/gbd-results/.

## Footnotes

^1^ https://www.healthdata.org/
